# Consistency of reports of violence from northern Rakhine state in August 2017

**DOI:** 10.1186/s13031-022-00453-1

**Published:** 2022-05-07

**Authors:** Sarah Trager, Jennifer Leigh, Andrea Woods, Parveen Parmar, Agnes Petty, Rohini Haar, Chris Beyrer

**Affiliations:** 1 Johns Hopkins Center for Public Health and Human Rights, Baltimore, MD USA; 2 Physicians for Human Rights, New York, NY USA; 3 Johns Hopkins Bloomberg School of Public Health, Baltimore, MD USA; 4 Clinical Emergency Medicine, University of Southern California, Los Angeles, CA USA; 5 School of Public Health, University of California, Berkeley, Berkeley, CA USA

**Keywords:** Rohingya, Myanmar, Consistency, Triangulation, Validation, August 2017, Violence

## Abstract

**Background:**

In August 2017, Myanmar’s Armed Forces, the Tatmadaw, launched an orchestrated attack on hundreds of Rohingya-majority villages in northern Rakhine state. This study seeks to validate the consistency of previous reports of violence against the Rohingya people in the region carried out by the Tatmadaw, Border Guard Police, and Rakhine villagers in the late summer and early fall of 2017.

**Methods:**

Internal validation data is from a three-armed study. Data analyzed in the external triangulation was sourced through a literature review of known, publicly available surveys and interviews. Both sets of data documented instances of violence against the Rohingya people in northern Rakhine state during the late summer and early fall of 2017. Consistency was evaluated across five indicators of violence: arson, presence of mass graves, reports of sexual violence and human injuries, as well as human fatalities, across 611 locales in northern Rakhine state. Further analysis was conducted to measure consistency of reports by locale and across locales by indicator.

**Results:**

Overall, an internal validation of 94 hamlets found that 98% of these locales were consistent across at least four of the five indicators (80% + consistency). Arson and reports of human injuries were the most consistent indicators across locales (100% and 99% consistency, respectively) and sexual violence was the least consistent indicator, with 84% of participating locales exhibiting consistent reports of sexual violence between the qualitative and quantitative data. Similarly, an external validation of 57 locations found that 50 of the 57 locations (88%) were consistent across indicators. Arson was the most consistent across sources (96%), whereas source agreement across locations was the least consistent for reports of sexual violence (58%).

**Conclusion:**

The government of Myanmar has denied involvement in the 2017 attacks on Rohingya communities in northern Rakhine state and purports that reports of the violence and destruction are overstated. However, consistent reporting from multiple sources on the same locales clearly underscores the veracity of the evidence documented, both by investigative groups and as recounted by Rohingya survivors of violence. It is our hope that this cataloging and comparison of available data, along with this study’s assessment of its consistency, will aid ongoing accountability efforts.

## Background

The situation concerning the Rohingya people has commanded international attention in recent years due to the violent persecution and genocidal campaigns perpetrated against them by the Bamar Buddhist-majority government of Myanmar, including Armed Forces, Border Guard Police, and others. For decades, the Rohingya people, a Muslim ethnic minority who live predominantly in Myanmar’s Rakhine state, have sought restoration of legal recognition and citizenship in a country formed on the land that has been their ancestral home [1]. Since 1948, with the de-colonization of Myanmar, the Rohingya people have gradually lost their political and civil rights; most notably, in 1982, their disenfranchisement was further legalized through the Citizenship Act, which denied the Rohingya people their Myanmar citizenship and all of the rights afforded to those recognized as citizens there.

The most recent wave of violence began in October 2016, when Myanmar’s military retaliated after the Arakan Rohingya Salvation Army, a Rohingya non-state armed group, carried out a series of attacks against local security posts [2]. Using its asymmetric arsenal to its advantage, Myanmar security forces led a disproportionate response on nearby Rohingya villages, inclusive of rape, arson, and murder. The violence waxed and waned over the following year, erupting in a widespread and systematic offensive across northern Rakhine state in August 2017 that expelled hundreds of thousands of Rohingya people from Myanmar [3, 4].

Documentation, including first-person testimonies, satellite photos, and quantitative and qualitative survey data, have captured the premeditated, indiscriminate, and extreme nature of the state-sponsored violence waged against the Rohingya. Entire villages were set on fire and families were forced from their homes and shot at point blank range or mutilated with household tools and swords. Women and young girls were sexually assaulted, often in front of their relatives, and property was looted and ransacked [5–7]. In response to the violence, more than 700,000 Rohingya people fled to neighboring Bangladesh and live in what is now the world’s largest refugee camp [8].

Human rights organizations, multilateral organizations, and watchdog entities have conducted a wide range of assessments of the 2017 assault against the Rohingya. Physicians for Human Rights (PHR) has assessed, monitored, and reported on this humanitarian crisis extensively, including a detailed account of the Chut Pyin massacre, documentation of sexual assaults, and reports from quantitative and qualitative investigations [3, 9–13]. Similar reports have been published by Amnesty International, Fortify Rights, Human Rights Watch, Médecins Sans Frontières, and *Reuters* [4, 6, 7, 14]. Estimates of deaths from the 2017 campaign range from 6700 to 7800 [13, 15].

Despite the existing testimonies and physical evidence of the systematic violence targeting the Rohingya people, there remains a need to collate available data in order to present a comprehensive case for the prosecution of perpetrators of this violence. This study aims to assess the breadth and consistency of available data on the atrocities inflicted on the Rohingya, by both internally validating data collected by PHR in 2018 and comparing it to data sourced from other organizations between 2017 and 2020. The findings presented here strengthen and validate the existing evidence, and it is our hope that they can be used by international accountability mechanisms to help secure restorative justice for the Rohingya.

## Methods

In 2018, PHR conducted a retrospective study with Rohingya refugees in Bangladesh to capture the experiences of people from all Rohingya hamlets in northern Rakhine state whose populations had been displaced to Bangladesh. The study had three arms: (1) a quantitative hamlet-level survey, (2) qualitative in-depth interviews with hamlet leaders, and (3) clinical evaluations of survivors to document physical sequelae of violence. Each of these arms have been documented in prior publications and are summarized below [11–13].

The study was conducted in the Rohingya refugee camps in Ukhiya and Teknaf upazillas in Cox’s Bazar District, Bangladesh. All of the respondents had arrived from Myanmar by October 2017, and all had previously resided in northern Rakhine state.

**Fig. 1:**
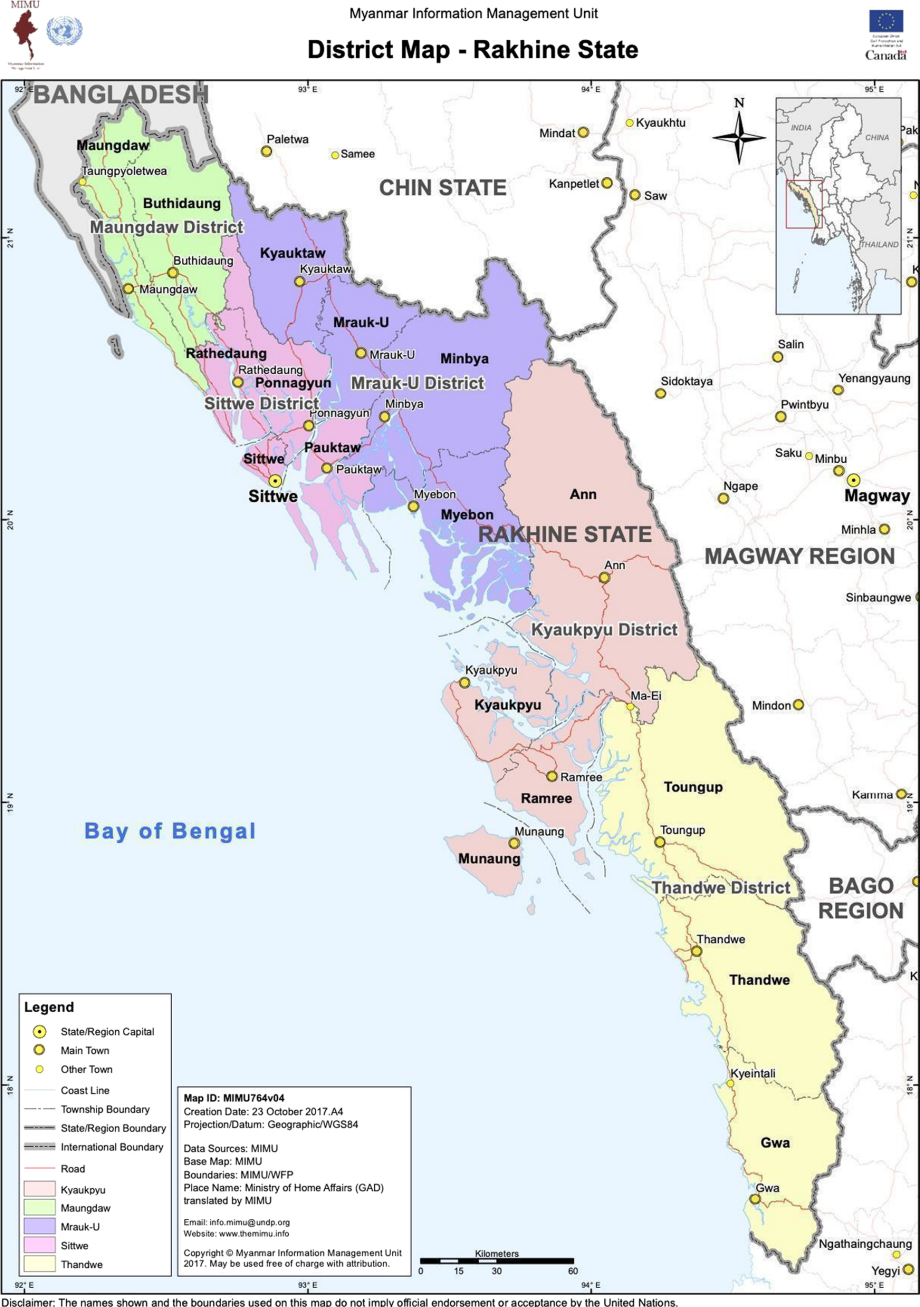
Map of townships in Rakhine state, Myanmar. Source: Myanmar: District Map—Rakhine State. (2017). Myanmar Information Management Unit. https://reliefweb.int/map/myanmar/myanmar-district-map-rakhine-state-23-oct-2017-enmy

**Fig. 2:**
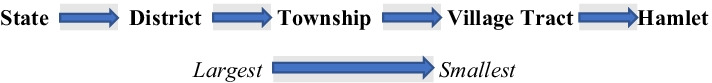
Relationship between administrative divisions in Myanmar. *Note* Northern Rakhine state is comprised of Maungdaw District, along with parts of neighboring Sittwe District. Within Maungdaw District, there are three townships: Rathedaung, Buthidaung, and Maungdaw. Each township has between 80 and 100 village tracts, which are each comprised of a group of villages, known locally as hamlets

An overview of the administrative divisions in Myanmar is necessary to understand the research methods. The majority of Rohingya who lived in Myanmar prior to August 2017 resided in Maungdaw District, which, along with parts of neighboring Sittwe District, is generally referred to as northern Rakhine state (Fig. 1). Within this District, there are three townships: Rathedaung, Buthidaung, and Maungdaw. Each township has between 80 and 100 village tracts, which are each comprised of a group of villages, known locally in English as hamlets. A visual depiction of these administrative divisions is below in Fig. 2.

### Data collection

In the quantitative arm, hamlet-level data was collected between May and July 2018 from 604 leaders of hamlets and urban wards in northern Rakhine state (Maungdaw, n = 346; Buthidaung, n = 229; Rathedaung, n = 29) [13]. In the Myanmar administrative system, hamlet and ward leaders are responsible for the regular reporting of local population data to the Myanmar government. These same individuals assumed leadership roles during the displacement to Bangladesh and continue to represent their former communities in the refugee camps. Based on this and through extensive consultation with the Rohingya refugee community and camp leadership, these leaders were determined to be representative interviewees for reporting on what happened to villagers before, during, and after flight to Bangladesh.

The surveys and in-depth interviews were conducted privately by trained Rohingya refugees representing each of the three affected townships from northern Rakhine state. Consenting participants were asked to report the frequency, scale, and type of violence experienced by the residents of their respective hamlets/wards during the offensive of August 2017 and their subsequent journey to Bangladesh [13]. If respondents reported a certain threshold of violence in their hamlet (10 or more deaths of villagers, mass rape, and/or witnessing of mass graves in their hamlet or during displacement), the respondent was invited to participate in an in-depth interview.

One third of quantitative survey respondents qualified for in-depth qualitative interviews. This was a higher number than anticipated, and due to resource constraints (e.g., time and money), qualitative interviews ended early. Interviews were conducted with 45% of those hamlet leaders who qualified, for a total of 88, with respondents from hamlets across all three townships of northern Rakhine state (Buthidaung, n = 42; Maungdaw, n = 34; Rathedaung, n = 12) [11]. The semi-structured interviews documented first-person testimonies of the violence and destruction that occurred within each respondents’ community, including attack details, reasons for fleeing, and experiences living in the settlement camps, among others [11].

The clinical evaluation arm documented physical injuries sustained by Rohingya survivors. Respondents were selected using purposive and snowball sampling to identify candidate participants who had experienced violence as evidenced by their physical scars or disabilities. Evaluations were completed among 101 individuals from 31 hamlets (Buthidaung, n = 7; Maungdaw, n = 19; Rathedaung, n = 5) by physicians trained in clinical evaluations during several visits from December 2017 through July 2018 [16]. Individual interviews were conducted during the evaluation to corroborate examination findings with geographic location and type of violence. Data from three individuals were unable to be location-matched based on available location data [12].

### Analysis

#### Internal validation

Internal validation was conducted if data existed for the same hamlet from at least two study arms (i.e., quantitative, qualitative, or clinical). Key indicators of interest were reviewed for comparison, including: arson, presence of mass graves, sexual assault, injuries, and fatalities. Given that measures varied in their structure across each arm (for example, closed-ended measures in the quantitative survey vs. open-ended text for in-depth interviews), a binary classification (yes/no) was created of whether each of the above indicators was reported for a specific hamlet or ward in each study arm.

Analyses were then conducted to assess whether indicators across the three study arms were consistent. Consistency was defined as the presence of a positive indicator across two or more internal data sources. While the quantitative survey and qualitative interview arms collected hamlet-level event data, the clinical interviews collected individual-level data about experiences of violence and physical and mental health outcomes. Thus, events included in those clinical interviews should generally be captured in the quantitative and qualitative data, but events reflected in the quantitative and qualitative data sources might not be documented in any particular individual narrative. The quantitative survey data is the most comprehensive, as respondents were prompted to select a response from pre-specified, closed-ended options for each of the indicators for comparison. Conversely, the qualitative interview was semi-structured, and the clinical evaluation was unstructured. Thus, a negative indicator in the qualitative or clinical data was not defined as inconsistent with a positive indicator in the quantitative data; however, a negative indicator in the quantitative data that was positive in the qualitative data or clinical evaluation is considered contradictory. The inclusion of three methods of data collection resulted in a broad base of comparable data, with more nuanced detailed narratives at both the hamlet and individual levels. Since the quantitative study arm was most comprehensive and had the largest sample (N), it served as the control method for comparison; it was equally as important to perform qualitative and clinical measures to capture data that may have been missed by the yes/no format of the quantitative survey.

#### External validation and triangulation

**Fig. 3:**
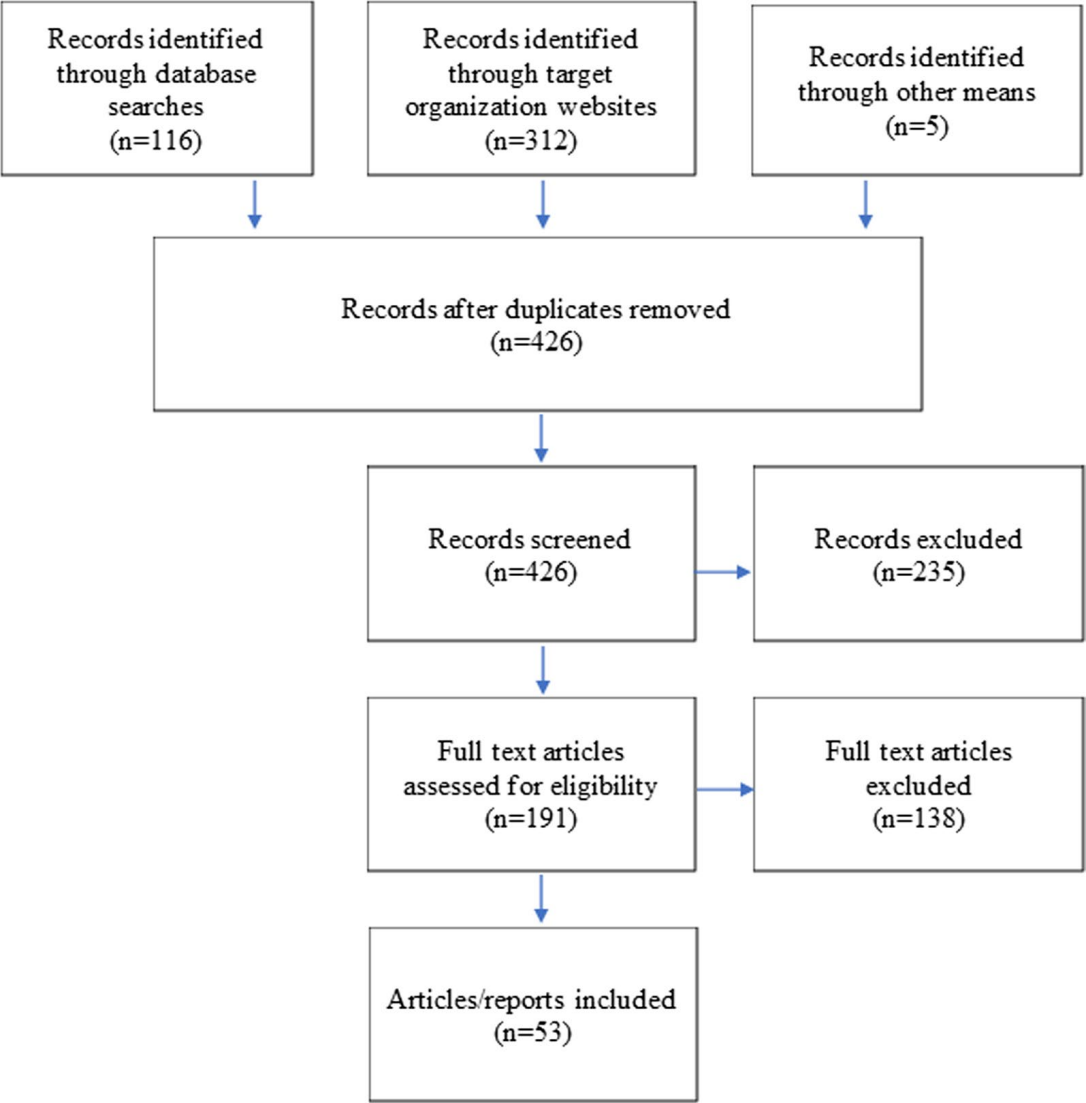
Prisma flow diagram of literature review for external triangulation

External validation and triangulation were also conducted with data from other publicly available data covering the 2017 violence in Rakhine state. A literature review was conducted to identify and extract comparable data. This approach was modeled after the PRISMA framework (Fig. 3).

An initial group of documents was collected from human rights organizations and other trusted organizations known to have reported on the violence against the Rohingya. These sources were chosen for their recognition as foremost human rights organizations, with widely respected and ethically sound reporting methods. The majority of the external data represented in this report is from one of nine main sources. The types of data collected varied across sources. Qualitative interviews were conducted by Amnesty International, Fortify Rights, Human Rights Watch, and Médecins Sans Frontières. Additional survivor testimony was reported by the *Associated Press, BBC News, Reuters,* and the UN’s Independent International Fact-Finding Mission on Myanmar (FFM). Extensive collections of satellite imagery and other remote sensing data were published by Amnesty International, Human Rights Watch, Reuters, the Women’s Refugee Commission, and the UN’s FFM. Several of the organizations utilized a mixed-methods approach to document these human rights abuses. For example, Amnesty International used NASA satellite imagery and environmental sensor data to detect evidence of fires in Rohingya hamlets across northern Rakhine state [17]. Additionally, it conducted both clinical examinations and qualitative interviews [18].

The websites of these organizations were reviewed for relevant publications and searched using key terms, including “Rohingya,” “Rakhine,” and “August 2017”. In total, 312 publications documenting the 2017 violence in northern Rakhine state were identified from these sources.

Additional literature sources were identified using the search engines Pub Med, Lexis Nexis, and Google. Key word searches included the terms “Rohingya” and “August 2017” with or without “Myanmar.” These searches yielded an additional 116 publications. Sources were also identified through citation mining, which contributed five further publications. Altogether, 426 unique records were identified, including articles, testimonies, comprehensive reports, qualitative interviews, quantitative health surveys, narrative journalism, and satellite imagery and other remote sensing data.

An initial rapid screening of titles and abstracts for relevance excluded 235 records, leaving 191 articles whose full text was assessed for eligibility (see Fig. 3). Literature was assessed for inclusion using the following criteria: accounts of violence perpetrated in the second half of 2017 in northern Rakhine state against the Rohingya; findings reported from primary data collection; published in English; data was available at the hamlet or village tract level; document was published and publicly available. The criterion by which sources were most frequently excluded was ‘failure to include primary data’.

Once relevant external literature sources were identified and extracted for triangulation, they were reviewed for village tract- or hamlet-level data that reported the following five key indicators: (1) arson, (2) presence of mass graves, (3) sexual violence, (4) human injuries, and (5) human fatalities. Each instance of violence that was reported in a specific administrative division was recorded. Qualitative interviews, testimonies, videos, and quantitative data were considered for each indicator. Satellite images and other remote sensing data provided information on arson. In all cases, the presence or absence of an indicator in a given source was assigned a binary (yes/no) designation for that indicator.

Standardization and alignment of locale names were required before data could be compared across sources. Due to the absence of an official list of hamlet and village tract names in both Burmese (the official state name), and Rohingya (a locally used name), a customized list was generated based on the Myanmar Information Management Unit (MIMU), input from non-governmental organizations which had operated in Rakhine state, and input from Rohingya research staff. Transliteration of Burmese and Rohingya names into English followed best practices established by local researchers and referenced either the MIMU or predominant spellings in existing Rohingya literature.

Rohingya is an oral language that does not have a written form. Because there is no standardized method for transliterating either Burmese or Rohingya words into English or Burmese script, this often results in an array of spellings for the same word. As a result, the spelling of geographic location names (i.e., township, village tract, hamlet) varied based on how they were recorded by individuals ranging from trained Rohingya data collectors to temporarily resident foreign journalists. This was especially true for data collected by different organizations, but even within the PHR data, there was sometimes variation between each of the three study arms. For example, “Pa Da Kah Ywa Thit” (clinical), “Pada Kar Ywa Thit” (quantitative), and “Ba Da Ga Ywa Thit” (qualitative) are different transliterations for the same hamlet. For Burmese names, the English spelling used was that designated by MIMU. For the Rohingya names, English spelling was standardized based on input from Rohingya researchers and generally following the predominant spelling in the literature.

Some locations were incorrectly identified in the open-source literature. In some cases, village tract names were presented as hamlet names, hamlet names that occur in multiple village tracts were given without specifying to which village tract they were referring, or the hamlet named was not part of the village tract to which that document assigned it. If a location was unclear due to mismatched hamlet and village tract information or alternative spellings, the location information was confirmed with the source organization, if possible. Most locales were readily identified, with only eight locales—representing 2% of the 348 locations specific accounts identified—for which a match could not be found. These eight locales, from five publications, were excluded from the comparative analysis, as there was no comparison data.

Data was then aggregated at the hamlet level. For information that identified the village tract but not the hamlet, data was aggregated at the village tract level. The aggregated data for an indicator of interest were presented as a fraction, with the numerator representing sources positive for that indicator (i.e., “yes” or present), and the denominator representing the number of sources potentially reporting on that indicator. For example, if five of the six sources reporting violence in Ah Htet Nan Yar hamlet identified the presence of arson, the arson indicator for Ah Htet Nan Yar would read 5/6.

Because most of the reports by other organizations presented only partial accounts or quotes from respondents and were not intended to be comprehensive accounts of occurrences in a particular hamlet, the PHR data was considered the baseline for comparison (e.g., internal data). External data was considered consistent with internal data if the internal indicator was positive and any of the external sources were positive, or if the internal indicator was negative and all of the external sources were negative. It is important to note that while a positive indicator signifies that the event in question was reported at that location, a negative indicator means only that it was not reported, rather than that it definitively did not occur.

Following aggregation, external data was assessed for consistency by locale and by indicator. For each locale, external data was compared to internal data for each of the five key indicators. Findings for a particular location were considered to be consistent overall if greater than 50% of the indicators were consistent (e.g., three or more of the five key indicators). Consistency was also assessed by indicator, across all locations, by calculating the proportion of hamlets for which sources reported consistently for that indicator.

Results are presented only in aggregate, without identifying the findings of each source individually. This is to protect the confidentiality of survey and interview participants who were hamlet leaders and thus identifiable by the hamlet name [13]. For that reason, PHR has not previously published findings at the hamlet level, and herein hamlet-level data is only presented in aggregate when there are at least two other sources. In order to ensure that we may continue to protect the identities of our subjects, the only hamlet-level data provided here is reporting consistency between study arms (Tables 1, 3, 4) and does not indicate whether or not the respondent reported instances of violence within a particular hamlet.

**Table 1:**
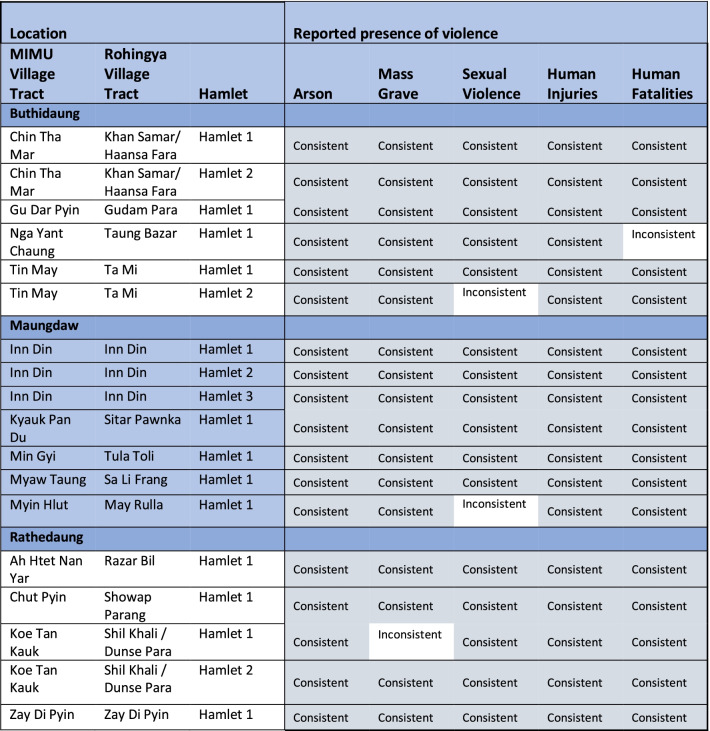
Internal validation: consistency across all three study arms of reported violence perpetrated against hamlets in northern Rakhine state in 2017

Consistency was defined as the presence of a positive indicator across two or more study arms (quantitative, qualitative, or clinical). A negative indicator in the clinical or qualitative data was not defined as inconsistent with a positive indicator in the quantitative data; however, a negative indicator in the quantitative data that was positive in the qualitative data or clinical evaluation is considered inconsistent

Color in table is to differentiate between townships

For two locations—Maung Nu and Min Gyi—where major events occurred and for which there are a high number of reports, more detailed analysis was conducted (Tables 3, 4), including numbers of injuries and fatalities, perpetrators, and types of violence. In these instances, we presented analysis at the hamlet level, but did not disclose which source reported which data so that a specific respondent’s reports could not be identified.

### Ethical review and human subjects considerations

The PHR Ethics Review Board (ERB) provided full ethical approval for this study. Because no formal Rohingya body exists that could serve as a review board, PHR held a community consultation with Rohingya leadership before administration of the qualitative and quantitative components of this work, to obtain their input, feedback, and approval. PHR’s ERB reviewed and approved the clinical arm. All study participants underwent an informed consent process in Rohingya language before participating in any of the study arms.

In order to protect respondent confidentiality and protect them from possible recrimination from the Myanmar government, no identifying information was recorded beyond hamlet of origin and leadership role of each respondent. Data was collected on password-protected tablets and transferred for storage on secure password-protected and encrypted servers. The research team discussed risks of this survey and their corresponding mitigation plans with all respondents, the Rohingya data collection team, and Rohingya leadership during community consultations.

## Results

### Internal validation

Data collected through the PHR study was internally validated across the three arms to establish a baseline level of consistency. Data was available from at least one of the three study arms for 611 locations. Internal validation was performed for locations for which data was available from at least two study arms, totaling 94 hamlets: 34 in Buthidaung, 48 in Maungdaw, and 12 in Rathedaung, of which 18 had available data for comparison across all three study arms.

Evaluating consistency based on the five key indicators reported, 67 hamlets (71%) were consistent across all five indicators. Twenty-five (27%) were consistent for four of five indicators, and two (2%) were consistent for three of five indicators. Arson was the most consistent. Though there were 14 hamlets for which the quantitative data was positive and the qualitative data was negative (which we consider consistent), there were no cases of the reverse. Sexual violence was the least consistent indicator, with 79 of the 94 hamlets (84%) exhibiting consistent reports of sexual violence between the qualitative and quantitative data. This was the only indicator in which there were more cases of the quantitative indicator being negative when the qualitative indicator was positive than the reverse. Reporting on mass graves was consistent in 95% of hamlets and reports of fatalities in 91%.

The subset of hamlets for which there is data from all three arms is presented below in Table 1. Of the 18 hamlets with data from all three study arms, 14 (78%) were consistent across all five indicators and the remaining four were consistent for four of five indicators. As in the full analysis, arson and reports of human injuries were the most consistent—in each case, there were no hamlets for which these indicators were inconsistent. Report of sexual violence was again the least consistent indicator, with inconsistency between the qualitative and quantitative data in two of the 18 hamlets. Reports were inconsistent for one hamlet for reports both of mass graves and of human fatalities. In all instances where inconsistencies were found, the quantitative survey data did not report the presence of these indicators within a particular hamlet, while the qualitative interviews from the same hamlet did report the presence of these indicators.

### External validation and triangulation

In total, 53 unique sources were identified through the comprehensive literature review with 348 total accounts, which provided data on the indicators of interest for 171 unique locations. Of those, 144 were at the hamlet level and the remainder at the village tract level.

Locations were included in this analysis if indicator data was reported in at least two external sources and the internal dataset. Data was available from at least two external sources for 63 of the 171 locations, of which matching internal data was available for 57 (Buthidaung: 12; Maungdaw: 39; Rathedaung: 6). For six locations, external data was available at the hamlet level, but internal data was unable to be matched—in two cases because there was no internal data collected from those hamlets and in four cases a match was not possible due to variations in hamlet names or use of wards instead of hamlet names.

Of the 57 matched locations, comparisons were made at the hamlet level for 29 and at the village tract level for 28. Comparisons were made at the hamlet level whenever possible. It is important to note that comparison across village tract-level data will likely be less consistent than similar comparisons between hamlet-level data because village tracts are comprised of multiple hamlets; one source reporting at the village tract level may capture an event in a particular hamlet, but a second source reporting on that village tract may not reflect findings from every hamlet in the village tract, and miss the event captured in the first report.

Thirty-eight of these 57 locations were compared across all five indicators. An additional 17 locations were compared on one indicator (arson), and two locations on two indicators (arson and sexual violence). Of these 38 locations, 10 (26%) were consistent across all five indicators, 14 (37%) across four, 9 (24%) across three, three (8%) across two, and two locations (5%) were consistent on only one indicator. The 17 locations that were compared only by the arson indicator were all consistent, and the two compared on arson and sexual violence were both consistent for the former but neither for the latter. As some sources did not report on all five indicators of interest, the denominator representing the number of sources discussing a particular location may not be the same for each indicator. For example, satellite photos only provide information on arson and two of the reports only focused on sexual violence. Village tract-level comparisons are presented in Table 2.

**Table 2:**
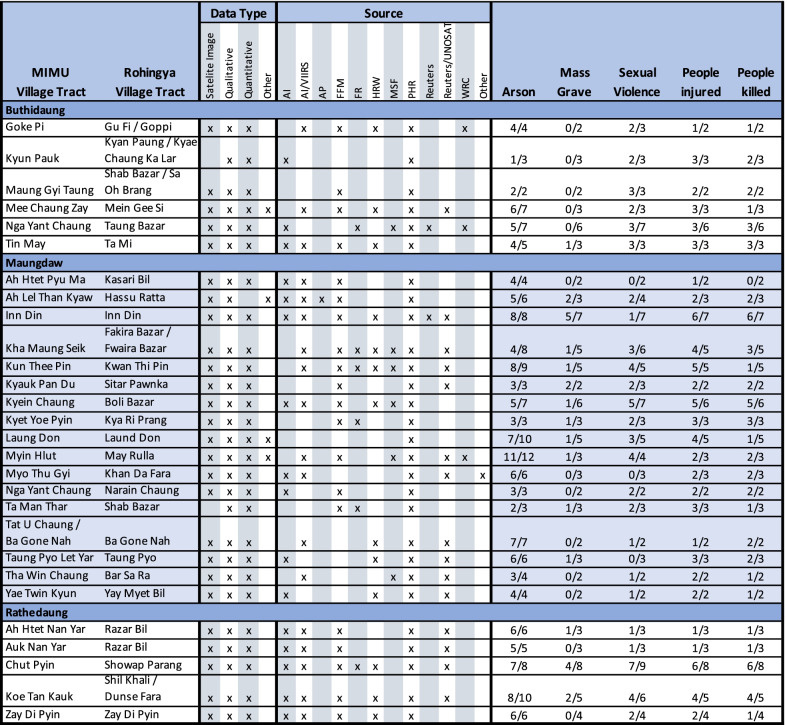
External validation of findings from village tracts with at least three sources of data

External validation presents findings by indicator for 28 village tracts from northern Rakhine state. At least three sources of data were available for each village tract. The fraction presented represents the number of sources reporting a positive (yes/present) indicator over the total number of sources for that location. The denominator is not consistent for all indicators because some sources only reported on specific indicators

Color in table is to differentiate between townships

**Fig. 4:**
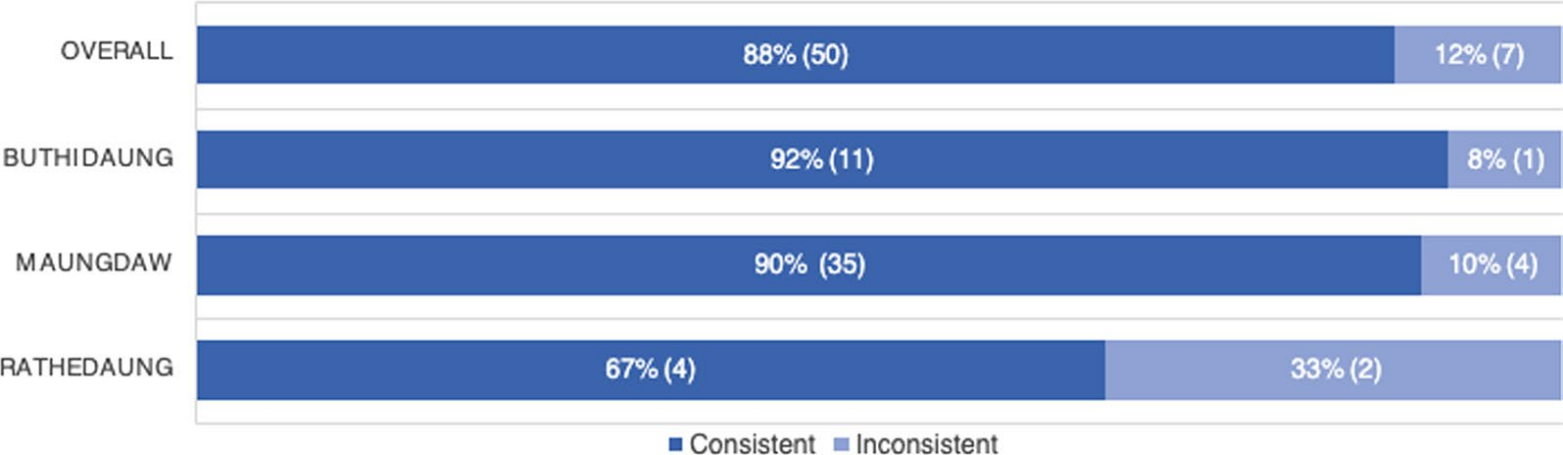
Overall external validity of reported violence in northern Rakhine state, 2017, by township. Note: Locations for which greater than 50% of indicators were consistent were considered to be consistent overall. Using this criterion, 50 of the 57 locations (88%) were consistent across indicators. Rathedaung locations were the least consistent at 67% (4/6), with Buthidaung 92% consistent (11/12) and Maungdaw 90% consistent (35/39)

To derive a sense of consistency across specific locations, locations for which greater than 50% of indicators were consistent were deemed consistent overall. Using this criterion, 50 of the 57 locations (88%) were consistent across indicators (greater than 50%). Rathedaung locations were the least consistent at 67% (4/6), with Buthidaung 92% consistent (11/12) and Maungdaw 90% consistent (35/39) (Fig. 4).

**Fig. 5:**
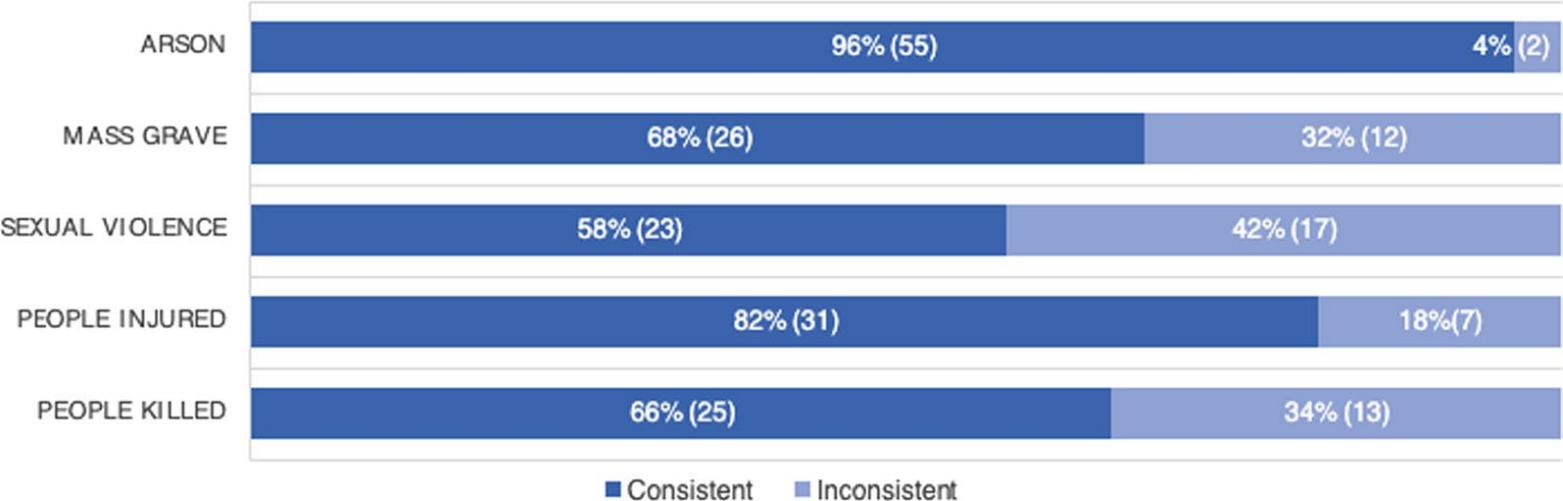
Consistency by indicator. *Note:* Data collected on arson was the most consistent across sources, as there was source agreement for 55 out of 57 locations (96%). Alternatively, source agreement was the least consistent for reports of sexual violence in 23 out of 40 locations (58%). Source agreement for reports of mass graves were consistent for 26 out of 38 locations (68%), for reports of human injuries in 31 out of 38 locations (82%), and for reports of human fatalities in 25 of 38 locations (66%)

When broken down by indicator, consistency varied. Data collected on arson was the most consistent across sources, as there was source agreement for 55 out of 57 locations (96%). Alternatively, source agreement was the least consistent for reports of sexual violence in 23 out of 40 locations (58%). Source agreement for reports of mass graves were consistent for 26 out of 38 locations (68%), for reports of human injuries in 31 out of 38 locations (82%), and for reports of human fatalities in 25 of 38 locations (66%) (Fig. 5).

In locations where events were widely reported by the media, more data was available. Notable events might include significant property destruction and/or mass killings carried out by the Tatmadaw in known Rohingya communities, which has been widely reported in the media. There were 10 locations for which data was available from more than five sources: Maung Nu/Mawnu Fara hamlet and Gu Dar Pyin/Gudam Para hamlet in Buthidaung; Min Gyi/Tula Toli hamlet, and Kyein Chaung/Boli Bazar, Myin Hlut/May Rulla, Kha Maung Seik/Fakira Bazar, and Inn Din village tracts in Maungdaw; and Chein Khali hamlet and Chut Pyin/Showap Parag and Zay Di Pyin village tracts in Rathedaung. For two of these locations, Maung Nu and Tula Toli, more detailed comparisons of findings are presented in Tables 3 and 4.

**Table 3:**
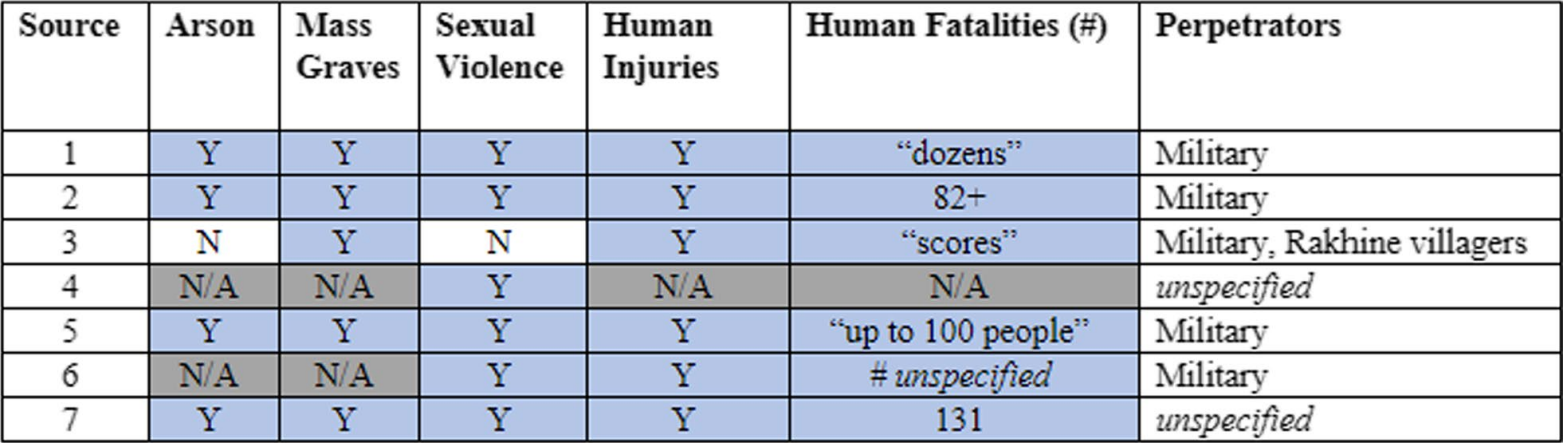
Source agreement chart for Maung Nu/Mawnu Fara hamlet, Buthidaung Township

External validation results of hamlet-level data for Maung Nu. To protect identities of each source’s subjects and the sources themselves, the data presented here is anonymized and, instead, listed numerically as sources 1–7. Source agreement for Maung Nu was highly consistent with an overwhelming majority of agreement within each indicator across all seven sources

Colors in table to differentiate between responses and for easier reading and analyzing trends. N/A = grey; Not present or "N"=white and Yes, present or "Y"=blue

**Table 4:**
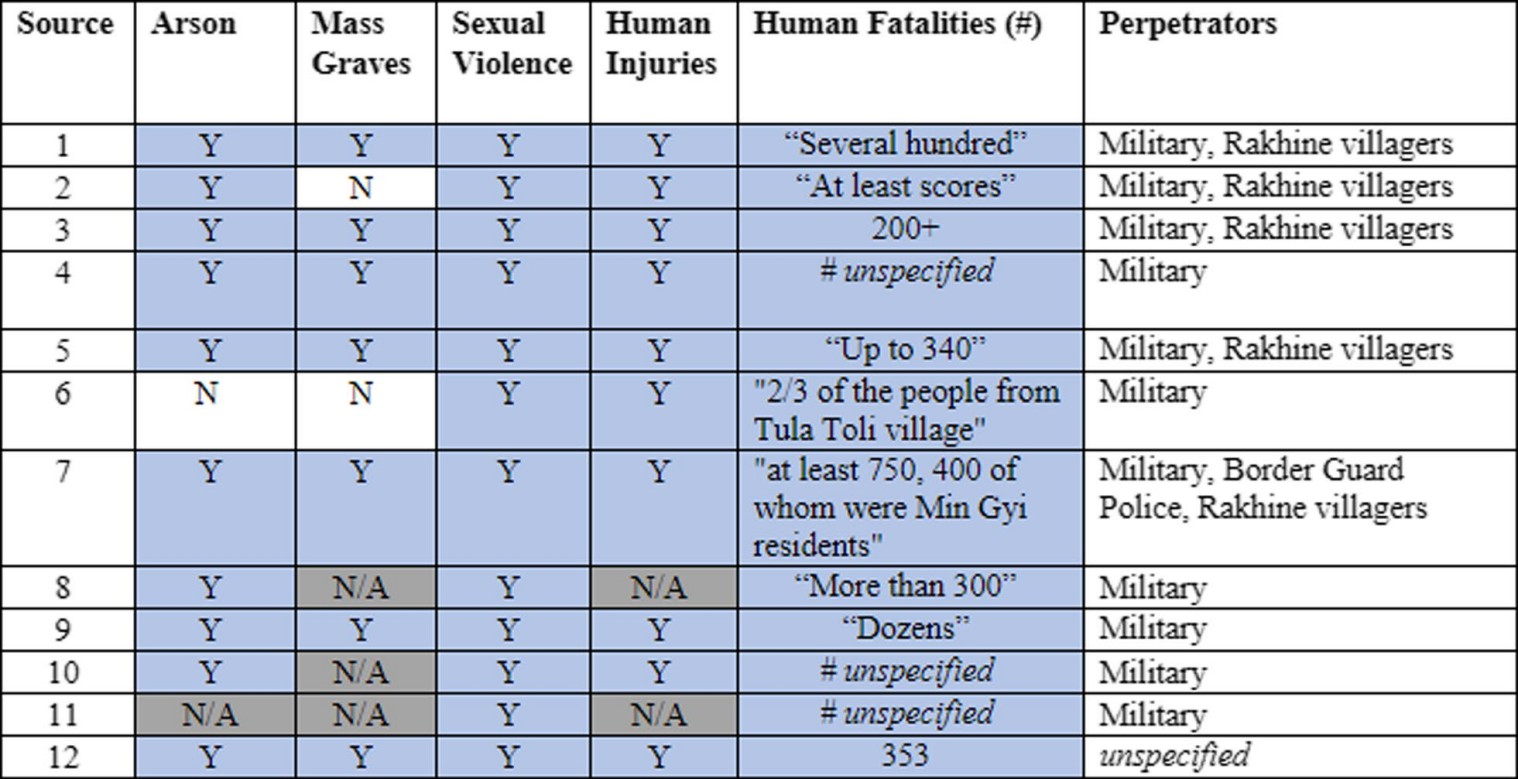
Source agreement chart for Min Gyi/Tula Toli hamlet, Maungdaw Township

External validation results of hamlet level data for Min Gyi. To protect identities of each source’s subjects and the sources themselves, the data presented here is anonymized and instead, listed numerically as sources 1–12. Source agreement for Min Gyi was highly consistent with an overwhelming majority of agreement within each indicator across all 12 sources

Colors in table to differentiate between responses and for easier reading and analyzing trends. N/A = grey; Not present or "N"=white and Yes, present or "Y"=blue

Source agreement for Maung Nu (Mawnu Fara) was highly consistent across indicators (Table 3). Of the seven reports, two focused explicitly on documenting sexual violence and therefore did not report on all indicators. Amongst the indicators for which there were reports, all sources reported the presence of a mass grave, human injuries, and human fatalities. Four out of five sources reported arson and six of seven reported sexual violence. Several accounts observed the Tatmadaw loading bodies wrapped in tarpaulin onto their military vehicles and boats to be transported elsewhere. Of those that reported perpetrator type, all five sources cited the military. Reports of the number of human fatalities ranged from dozens to 131. The types of violence reported were highly consistent in their reports of extreme brutality, including decapitation, dismemberment, and homicide by drowning.

Source agreement for Min Gyi (Tula Toli) was also highly consistent (Table 4). Twelve out of 12 sources reported both sexual violence and human fatalities in Min Gyi. Ten out of 10 sources reported human injury and 10/11 reported instances of arson. Seven out of nine reported the presence of a mass grave. The number of reported human fatalities in Min Gyi varied considerably, and some sources did not provide numerical estimates; however, those that did reported upwards of “several hundred” killed. One source cited “at least 750, 400 of whom were Min Gyi residents,” Like Maung Nu, the reported violence in Min Gyi was predominantly perpetrated by the military, but more sources here implicated Rakhine villagers in the abuses. In addition to reports of specific violence captured above, various sources also reported instances of looting and extreme brutality and homicide by drowning, burning, beating, decapitation, stabbing, and trampling. Most of the sources specifically mentioned people being burned alive and throats being slit.

## Discussion

This study collates and presents data on violence against Rohingya civilians in more than 600 hamlets and wards across all three townships of northern Rakhine state, representing very nearly all hamlets in northern Rakhine State with Rohingya inhabitants. While the exact proportion of hamlets with Rohingya inhabitants is unknown, it is estimated to be approximately two thirds of the total 912 hamlets MIMU reports (~ 600), notably higher than the 471 hamlets a Myanmar state official cited in September 2017 [19, 20].^1^ Similarly, there is no definitive listing of the hamlets that experienced violence in August and September 2017. The quantitative study described here is, therefore, the most comprehensive data collection effort undertaken. Of the village leaders from 590 hamlets and 12 urban wards included in that sample, 88% reported violence occurring in their hamlet. Lacking any other equivalent estimate with which to compare that finding, the triangulation reported on here serves to compare findings from locations where more than one source collected information in order to bolster and validate those findings.

This study is unique in identifying the locations and types of violence within each location based on three separate research methods as well as an extensive review of primary data from more than a dozen organizations. The internal validation examined location and indicator-specific consistency across the three study arms, whereas the external triangulation validated the consistency of PHR’s findings when compared to similar investigations by other organizations. Moreover, the internal validation affirms the accuracy of both the quantitative and qualitative data which were critical to this study when conducting the external triangulation. Performing both forms of validation further strengthens our findings and the veracity of the civilian-reported data from northern Rakhine state as a whole. The combination of internal validation and the external triangulation and validation demonstrated a high level of consistency in the data, underscoring the robustness of the available data, as well as the accompanying methods which can aid in further investigations.

Prior to this analysis, there was no accounting of existing data on the 2017 assault against the Rohingya. External sources covered a combined total of 171 unique locations, with the majority being reports of arson derived from satellite photos and remote sensing. PHR data covered 611 unique hamlets and wards, including 604 locations with extensive quantitative data. The compilation presented here, therefore, is a unique catalog of available data, which enables comparison of findings across accounts. The documentation of violence in so many hamlets across all three townships of northern Rakhine state also firmly underlines the widespread nature of the violence perpetrated against the Rohingya in this area. Although there were undoubtedly some accounts that were not captured in this review, this analysis includes the vast majority of primary data on the violence and provides an informative cross-section of available information.

That being said, it is also important to note that collecting any data in a humanitarian crisis is challenging. Settings of acute crisis present manifold impediments to implementing standardized research methods, collecting data from widespread areas, or from a large sample. Challenges include difficulty accessing interviewees who are mid-flight or displaced in refugee camps, lack of documentation of the ongoing conflict environment for reference and background, and in this case, visual evidence of the Myanmar Government actively covering up the brutality by bulldozing and rebuilding on devastated Rohingya hamlets [21]. Due to this challenging landscape, the combined evidence collected in this study may still fail to capture all of the violence that occurred during the timeframe of study.

### Consistency by indicator and method

Arson was the most common form of destruction and one of the easiest to observe, both by witnesses and by aerial and satellite photography, which may be why reports of arson were the most consistent across datasets both internally and externally. When compared with external sources, reports of arson were consistent for 55 of 57 hamlets (96%), the most consistent of the five indicators assessed by a notable margin. In both those cases, PHR data reported that arson had occurred, but neither of the two external qualitative sources available included information on arson. In no case where both PHR quantitative data and remote imagery was available was there inconsistency.

The least consistent indicator, both internally and externally, was reports of sexual violence. Internal hamlet-level data found reports of sexual violence to be consistent in 84% of hamlets, whereas reports of sexual violence from the external triangulation were much less consistent, congruous in only 58% of hamlets. There are several potential reasons for higher levels of discrepancy in the reporting of sexual violence. PHR respondents were all hamlet leaders (exclusively male) and may have been less aware of instances of sexual violence perpetrated against women. Furthermore, there is a cultural reluctance to speak about such a stigmatizing topic amongst the Rohingya, particularly in cases where sexual assault against men has occurred. Several interview respondents discussed fear of disclosing sexual violence due to religious and social stigmas surrounding sexual impurity, regardless of whether the act was consensual or forced.

With respect to method, the authors found that three external sources which focused exclusively on documenting instances of sexual violence did report instances that would not otherwise have been recorded [22–24]. Their approach differed in that they prioritized interviewing victims and witnesses of sexual violence, referred to them by local stakeholders (e.g. NGOs, think tanks, advocates, etc.). At least one of these three reports documented sexual violence in two thirds of the total locations reporting sexual violence [23]. The specific attention these investigations paid to interviewing victims and witnesses of sexual violence resulted in them being more likely to capture instances of this type of violence and to verify its occurrence through multiple reports of the same instance.

The discrepancy in consistency by indicator may have been impacted too by the difference in how each indicator presents. For example, arson can easily be identified by smell, sight (smoke or fire) through personal observation or remote satellite imaging, and the presence of charred destruction of nature and/or property long after the incident occurred; conversely, when documenting reports of sexual violence, researchers must rely on interviewees to have been made aware of the incident through personal experience, observation, or disclosure and be forthcoming about sharing this information. As indicated earlier, when measuring sexual violence, therefore, consistency of reports may be more vulnerable to where or from whom this data is collected as well as the social stigmas around this type of violence, potentially leaving instances to go unreported.

Interestingly, reports of human fatalities also had lower levels of consistency. Amongst the locations where the external triangulation and validation found an inconsistency, there were 13 cases where PHR reported death but no external source did, whereas there were no cases of the reverse. The inconsistency may be related to the high proportion of villagers who fled during and following the violence. During the chaotic flight from northern Rakhine state to Bangladesh, individuals who observed violence may not have been able to ascertain whether injured civilians survived or not. PHR data was collected several months after respondents arrived in Bangladesh; at this point, hamlet leaders had time to take accounting of the individuals displaced from their hamlet and ascertain the fates of fellow villagers and family members. Even at that time, some uncertainty remained, as some individuals remained in northern Rakhine state, some were reported missing, some sought asylum in other countries, and not everyone reconnected in the expansive camps.

As with fatalities, PHR data was more likely to report the presence of mass graves. There were 10 locations for which PHR reported a mass grave, without similar reports from any external sources. There was one location for which PHR reported no grave, and one of four external sources reported one. In this instance the reference was to a pile of burned bodies. In this case and others, inconsistent reporting may be due to confusion regarding the definition of a mass grave, which could have resulted in misclassification and under-reporting. Although there is no consistent international definition of a mass grave, interviewers were instructed to use the definition of three or more victims of execution buried or burned together in a common site. Because there were multiple reports of dead bodies being piled up, thrown in ponds, or gathered in houses that were burned, some respondents may have been unclear about whether or not to report such incidents as mass graves. Additionally, many respondents left the scenes of violence immediately, resulting in fewer witnesses to the aftermath, including the disposition of bodies in mass graves. This is another indicator for which remote sensing data may prove useful, as it can be pulled for multiple time points, including after displacement.

### Recommendations

Reflecting on this analysis, and the comparisons discussed here, we have identified several recommendations for other human rights documentation research oriented towards ensuring that human rights data is collected and analyzed as rigorously as possible for use by accountability mechanisms.

To better gauge the magnitude of violence, against a specific population, by location, it would be useful to have a listing of Rohingya hamlets, standardized number and spelling, and whether each was affected. This goal could be achieved by cooperation with a central body such as the HRC, or body like the IIMM if one is established, which could create/update a list on its website, and invite human rights organizations to submit location specific data on a few key indicators.

Furthermore, the lack of specificity in identifying locations was also a challenge. Many documents failed to differentiate between village tracts and hamlets. In some places, hamlets are like different neighborhoods of the same village, but often, they are separate villages in the same area, but separate enough that they may have had different experiences. Because not all sources clarified which administrative level they were naming, it was sometimes difficult to understand the comparability of data. We therefore recommend including as much geographic specificity as possible when documenting human rights violations. As noted above, the incorporation of aerial and satellite photos and other remote sensing data is a useful addition to more traditional testimonies and interviews. Remote sensing data can capture wide areas, and potentially make temporal comparisons. We recommend the continued and further incorporation of these forms of data in human rights documentation. As it requires particular knowledge, access, and skills, we suggest both exploring training for human rights organizations, and also growing partnerships with organizations that perhaps aren’t traditional human rights organizations, but have these particular expertise.

With respect to maximizing documentation of sexual violence, we recommend making it a specific focus of inquiry, including specific questions about sexual violence in both qualitative and quantitative data collection, and seeking out respondents who are well suited to report such incidents.

The presence of mass graves is frequently an indicator of interest in settings of mass violence and atrocity. Currently, without a standard and consistent international definition of a mass grave, this leaves studies vulnerable to discrepancies in documentation. Therefore, we recommend the development and standard utilization of an international definition to help forestall undercounting or misidentification in future human rights documentation.

Finally, it is important to note that only the PHR survey data was collected with the intention of providing systematic and population-based evidence of the violence that was perpetrated against the Rohingya in hamlets across all three townships of northern Rakhine state. Most other data sources were not meant to be comprehensive accounts, generally focusing on a few locations or collating survivor testimonies. Therefore, the failure of an external data source to include a report on one of the key indicators does not reflect that the event did not occur, rather that the account did not document it. No external sources specifically documented the absence of specific events. Applying epidemiologic methods to human rights documentation can help to fill some of the gaps, by contributing to more systematic, comparable, and comprehensive data collection. In doing so, this may serve to bolster the more in-depth testimonial accounts which are more commonly documented.

## Conclusion

The government of Myanmar has denied involvement in the 2017 attacks on Rohingya communities in northern Rakhine state and purports that reports of the violence and destruction are overstated. This analysis clearly demonstrates the extensive evidence base that exists to document the violence. Multiple organizations, utilizing multiple qualitative and quantitative methodologies, have independently recorded events, including destruction of homes and property, fatal and non-fatal violence against civilians, sexual assault of both men and women, and extrajudicial killing. The available data covers a wide area, stretching across all three townships of northern Rakhine state. Data is available from more than 600 geographically distinct locations, with three or more sources documenting more than 50 of them. Consistent reporting from multiple sources on the same locales further underscores the veracity of the evidence documented, both by investigative groups and as recounted by Rohingya survivors of violence.

Designing, conducting, and analyzing a mixed-methods assessment of human rights violations of this scale takes considerable resources, as do external validation methods, and challenges the timely dissemination of results for advocacy purposes. Nonetheless, this analysis demonstrates that post-hoc validation can be conducted and provides further evidence of the strength of data that is independently collected by multiple organizations.

The internal validation and the external triangulation and validation found that the data is highly consistent both across study arms within each hamlet and across various sources for each unique hamlet or village tract location. The overall level of internal and external consistency underscores the robustness of the extensive data collected by both PHR and other human rights organizations.

We recommend that future efforts to document human rights widespread violence and atrocities, much like the PHR documentation efforts, consider the inclusion of quantitative data collection methods, to provide a different picture and enable collection of comparable data across a range of locations. Remote sensing data can also be a valuable addition.

It is our hope that this cataloging and comparison of available data will aid accountability efforts, such as those ongoing at the International Criminal Court and the case brought forth by The Gambia at the International Court of Justice. The documentation of evidence from so many locations, and the similarities of experiences in those locations support allegations that the violence was both widespread and systematic—two of the criteria for defining actions as crimes against humanity [25]. Informed by both internal validation and extensive triangulation of data documenting violence against the Rohingya from the 2017 attacks, we are confident that the breadth, consistency, depth, and rigor of this documentation underscores the strength of the evidence base documenting the 2017 attacks.

## Data Availability

The data is available from the corresponding author on reasonable request, with appropriate redactions. Due to concerns about identification of respondents, hamlet level-data will not be shared publicly. No identifying information on respondents was collected beyond their hamlet of origin and leadership role; however, even that information may narrow the pool of potential respondents sufficiently to constitute a risk of identification, and, consequently, the real threat of reprisal from the Myanmar government or military following any repatriation efforts to their homeland in northern Rakhine state. Therefore, the research team has decided not to release any data at hamlet level which includes the hamlet name. Data may be shared at village tract level if hamlet names and respondent roles are redacted.

## Abbreviations

PHR: Physicians for Human Rights
MIMU: Myanmar Information Management Unit
ERB: Ethical Review Board
FFM: Fact Finding Mission

## Footnotes

1 Note: The Myanmar official cited may have been referring to hamlets with *only* Rohingya inhabitants.
